# A drug-based model to predict hyponatremia in outpatients of a geriatric clinic

**DOI:** 10.1007/s00228-025-03890-y

**Published:** 2025-08-01

**Authors:** Anne Claire B. van Orten-Luiten, Elske M. Brouwer-Brolsma, André Janse, Renger F. Witkamp

**Affiliations:** 1 Division of Human Nutrition and Health, Wageningen University & Research, Wageningen, The Netherlands; 2 Nutrition & Healthcare Alliance, Ede, The Netherlands; 3 Department of Geriatric Medicine, Gelderse Vallei Hospital, Ede, The Netherlands

**Keywords:** Hyponatremia, Aged, Geriatrics, Outpatients, Polypharmacy, Inappropriate prescribing

## Abstract

**Purpose:**

Chronic hyponatremia in older people is associated with adverse outcomes including gait disturbances, falls, osteoporosis, fractures, cognitive impairment, and cardiovascular disease. Diagnosis in outpatient settings is challenging due to the non-specific nature of its symptoms. While hyponatremia is well-studied in hospitalized patients, little research has focused on outpatient settings. This study aimed to develop a drug-based model to non-invasively predict hyponatremia in older adults attending a geriatric outpatient clinic.

**Methods:**

Cross-sectional data from 2181 outpatients aged ≥ 55 were analysed using logistic regression. Polypharmacy, 27 specific drug groups, sex, age, and BMI were considered as potential risk factors. Predictors were selected using stepwise backward logistic regression for the complex model and LASSO regression for the simple model. Internal validation was performed through bootstrapping, and model performance was evaluated by constructing a receiver operating characteristic (ROC) curve.

**Results:**

The prevalence of hyponatremia was 10.5%, with higher occurrence in women. The complex model identified predictors including sex, age, BMI, polypharmacy, and 11 drug groups, achieving an area under the curve (AUC) of 0.75, [95% CI 0.72–0.79], indicating a reasonably good ability to distinguish between hypo- and normonatremia. The simple model, including only polypharmacy, had limited predictive performance (AUC = 0.64 [95% CI 0.60–0.68]).

**Conclusion:**

The complex, drug-based model predicts hyponatremia risk in outpatients of a geriatric clinic. Timely recognition may prevent inappropriate treatments for undiagnosed cases and associated harms. The model merits further development for clinical use.

**Supplementary Information:**

The online version contains supplementary material available at 10.1007/s00228-025-03890-y.

## Introduction

Hyponatremia, defined as plasma sodium concentration below 135 mmol/l, is a common electrolyte imbalance frequently diagnosed in older people. The estimated prevalence ranges from 6.9% to 8.0% among older visitors of primary care [1, 2]. Variation in estimates may be attributed to differences in population characteristics, laboratory techniques, and types of hyponatremia. At the physiological level, it is important to differentiate between true, secondary, and pseudohyponatremia. True or hypotonic hyponatremia is characterized by low serum osmolality due to a disruption in body water balance. The underlying causes are often complex and multifactorial, with advanced age, chronic kidney disease, and drug use being significant risk factors [3, 4]. Secondary or hypertonic hyponatremia arises from an excess of circulating glucose or other osmotically active solutes [5]. Clinically, low sodium levels in hyperglycemic emergency patients are only linked to mortality when adjusted for glucose levels [6]. Pseudohyponatremia, or normotonic hyponatremia, occurs in patients with severe hyperlipidemia or hyperproteinemia, where elevated lipid or protein levels lower the measured sodium concentration in total plasma. However, sodium concentration in the aqueous phase of the plasma remains unchanged. This laboratory artifact only occurs when sodium concentration is measured in diluted plasma [5].

Clinically, distinguishing between acute and chronic hyponatremia is essential, as speed of onset determines its clinical manifestation. Acute hyponatremia develops within 48 h and can cause life-threatening neurological symptoms. In contrast, symptoms of chronic hyponatremia are non-specific, including fatigue, dizziness, cognitive disturbances, unsteady gait, falls, and fractures [7, 8]. These symptoms are easily misinterpreted as an aggravation of an existing condition, a new medical condition other than hyponatremia, or as normal consequences of aging. Moreover, chronic hyponatremia has been associated with an increased risk of cardiovascular disease and mortality [9]. Despite this, chronic hyponatremia is an underdiagnosed condition, which may result in inappropriate treatments which fail to address the underlying electrolyte imbalance [10]. Moreover, while hyponatremia is well studied in hospitalized patients, little research has focused on outpatients. Therefore, the aim of this study was to develop a predictive model to estimate individual risk of hyponatremia, based on non-invasive and easy-to-measure variables in a population of Dutch older adults visiting a geriatric outpatient clinic. Given that outpatient evaluations are typically scheduled for non-urgent complaints such as cognitive impairment and falls, many cases may represent chronic hyponatremia, although this cannot be confirmed by our data.

## Methods

### Study setting

This study used a cross-sectional design based on data from the PanDeMics (Polypharmacy and Deficiencies of Micronutrients) study, including 3541 community-dwelling older adults attending the outpatient clinic of the Geriatric Department of the Gelderse Vallei Hospital in Ede, The Netherlands, between 3 January 2011 and 13 June 2016. The main indications for consultation were cognitive impairment or falls. Inclusion criteria for this study were age ≥ 55 years and available data on plasma sodium concentration, renal function (estimated glomerular filtration rate (eGFR)), body mass index (BMI), and drug use (N = 2762). Patients with severe chronic kidney disease or kidney failure (eGFR < 30 ml/min/1.73m^2^) were excluded. Additionally, users of antidiabetic medications were excluded to reduce the number of cases of secondary hyponatremia. Ultimately, data of 2181 patients were included in the statistical analyses.

### Measurements

Sodium concentrations were measured in non-fasting blood samples collected in lithium heparin tubes and analyzed through ion-specific potentiometry (V-Lyte Integrated Multisensor Technology, Siemens Healthcare Diagnostics Inc. Erlangen, Germany) in the Clinical Chemistry and Hematology Laboratory of Gelderse Vallei Hospital in Ede. The eGFR was calculated with the Modification of Diet in Renal Disease equation [11], based on creatinine concentration measured in lithium heparin plasma by colorimetry (ECREA method, Siemens Dimension Vista® System). During the baseline consultation, the physician inquired about smoking status (never, ever, or current) and alcohol use based on the Alcohol Consumption Index [12]. For calculation of BMI (kg/m^2^), height (cm) and weight (kg) were measured according to a protocol. Cutoff values of 21 kg/m^2^ for underweight (based on the MNA questionnaire) and 27 kg/m^2^ for overweight were applied [13]. All prescribed and over-the-counter drugs and supplements were registered based on (hetero)anamnesis, pharmacy medication lists and those from the referring physician. Patients were also asked to bring all their drugs and supplements to the consultation. All substances were coded according to the Anatomical Therapeutic Chemical (ATC) classification system [14]. Polypharmacy and severe polypharmacy were defined as the concomitant use of at least five and ten different ATC-coded substances, respectively. Drug groups included in the statistical analyses were proton pump inhibitors (PPIs), osmotically acting laxatives, multivitamins and minerals, vitamin D, calcium, potassium, vitamin K antagonists, platelet aggregation inhibitors, cardiac therapy, thiazide diuretics, loop diuretics, potassium-sparing diuretics, selective beta blockers, dihydropyridines, angiotensin-converting enzyme (ACE) inhibitors, angiotensin-2 antagonists, statins, thyroid preparations, non-steroid antiinflammatory drugs (NSAIDs), bisphosphonates, opioid analgetics, anilides (paracetamol), antiepileptics, antipsychotics, benzodiazepines, antidepressants, and adrenergic inhalants. This selection was based on previously reported associations with hyponatremia [4, 15] or a minimum usage rate of 5% in the study population. For further details on the study population and measurements we refer to previous publications [16, 17].

### Data analysis

Data were analyzed with SPSS statistical software, version 29.0.1.0. Patient characteristics were calculated for the total study population and the subgroups diagnosed with either hyponatremia or normonatremia (sodium ≥ 135 mmol/L) and expressed as mean (± standard deviation (SD)), median (interquartile range (IQR)), or frequency (%). Two regression models were developed, including sex, age, BMI, polypharmacy, and 27 pharmacologically active agents as potential predictors. Hyponatremia was defined as plasma sodium < 135 mmol/L. A first, complex model was constructed with the variables selected through stepwise backward logistic regression. Nagelkerke R Square was determined as a measure of goodness-of-fit. Internal validation was performed using bootstrapping assessing the robustness of the confidence intervals of the regression coefficients A second, simplified model was developed with the variables selected through least absolute shrinkage and selection operator (LASSO) regression. This technique reduces potential overfitting and improves model interpretability by selecting only the most impactful variables. The regression coefficients from both models were transformed into predictor risk scores by multiplying them by 2 and rounding the results to the nearest integer [18]. The resulting predictor risk scores ranged from −2 to + 3. The total risk score was calculated by summing the predictor scores and adding 11 to the constant ensuring a minimum total score of 1. Model performance was evaluated using the area under the receiver operating characteristic (ROC) curve (AUC), which quantifies the model’s ability to distinguish between normo- and hyponatremia. For this purpose, the individual probability P of hyponatremia was calculated using the formula P = 1/(1 + e^−logit P^) in which logit P was calculated as follows: logit P = constant + b_1_x_1_ + b_2_x_2_ + …. + b_k_x_k_; b_1_,.…b_k_ are the predictor risk scores. With these probabilities a ROC curve was created by plotting the true positive (TP) rate (sensitivity) against the false positive (FP) rate (1-specificity) to determine optimal model score cutoff values.

## Results

### Characteristics of the study population

The prevalence of hyponatremia was 10.5% in the total population, 7.6% in men, 12.6% in women. The prevalence of severe polypharmacy (concomitant use of at least ten ATC-coded substances) was observed in 30.0% of participants with hyponatremia, compared to 13.9% with normonatremia. For no polypharmacy (use of no more than four ATC-coded substances) these figures were 22.6% and 44.0%, respectively (Table 1). The most used drug class was proton pump inhibitors, with a prevalence of 50.1% (electronic supplementary material).

**Table 1:** Characteristics of 2181 outpatients of a geriatric clinic (mean (**± **SD), median [IQR], frequency (%))

Characteristic	Category	Total study population ^a^	Hyponatremia ^b^ (*n* = 230) ^c^	Normonatremia (*n* = 1951)
Sex (n)	men	902	69	833
	women	1279	161	1118
Age (yr)		78.2 (± 8.5)	81.4 (± 6.5)	77.8 (± 8.6)
	< 70	16.0%	4.8%	17.4%
	70–79	34.2%	32.6%	34.3%
	> 79	49.8%	62.6%	48.3%
BMI (kg/m^2^) ^d^		26.4 (± 4.5)	25.5 (± 4.2)	26.5 (± 4.6)
	< 21.0	8.9%	14.8%	8.2%
	21.0–27.0	51.1%	53.0%	50.8%
	> 27.0	40.0%	32.2%	41.0%
Albumin (g/l)		42.2 (± 4.2)	41.7 (± 4.5)	42.3 (± 4.2)
Sodium (mmol/l)		139 (± 3)	131 (± 3)	139 (± 2)
Potassium (mmol/l)		4.1 (± 0.4)	4.1 (± 0.4)	4.1 (± 0.4)
Glucose (mmol/l)		5.6 [5.2–6.2]	5.7 [5.2–6.4]	5.6 [5.2–6.2]
GFR (ml/min) ^e^	30–60	23.9%	20.0%	24.3%
MNA screening (score) ^f^		12 [11-13]	12 [10-13]	12 [11-13]
	0–7	5.7%	7.5%	5.5%
	8–11	29.4%	36.1%	28.5%
	12–14	64.9%	56.1%	66.0%
MMSE (score) ^g^		25 [21-28]	25 [22-27]	25 [21-28]
	0–18	17.9%	15.0%	18.2%
	19–24	30.0%	32.7%	29.7%
	25–30	52.1%	52.3%	52.1%
Pharmacologically active agents ^h^ (n)		5 [3-8]	7 [5-10]	5 [3-8]
- drug^h^ use (≥ 1)	yes	93.8%	98.7%	93.2%
- no polypharmacy (0–4)	0–4	41.8%	22.6%	44.0%
- polypharmacy (5–9)	5–9	42.6%	47.4%	42.1%
- severe polypharmacy (≥ 10)	≥ 10	15.6%	30.0%	13.9%
- dietary supplement ^i^ use (≥ 1)	yes	50.7%	62.2%	49.3%

^a^ missing (n): potassium 19, glucose 80, albumin 56, MMSE 87; ^b^ sodium blood level < 135 mmol/L; ^c^ missing (n): potassium 5, glucose 10, albumin 12, MMSE 16; ^d^ Body Mass Index; ^e^ Glomerular Filtration Rate; ^f^ Mini Nutrition Assessment; validated for 65 plus: n = 1617 assessments in a subpopulation of n = 1996 patients aged ≥ 65 years (missing MNA: n = 379); of which n = 179 is hyponatremic; (n = 1702 assessments in total population (missing MNA = 479)); ^g^ Mini Mental State Examination; ^h^ any ATC-coded substance (Anatomical Therapeutic Chemical classification); for use of specific ATC-coded substances see electronic supplementary material; ^I^ ATC-coded supplements A02AA, A11, A12, B03A-, B03B-, A16AA01,C10AX06, M01AX05, N05C, N06DX02

### Prediction of hyponatremia

**Fig. 1:**
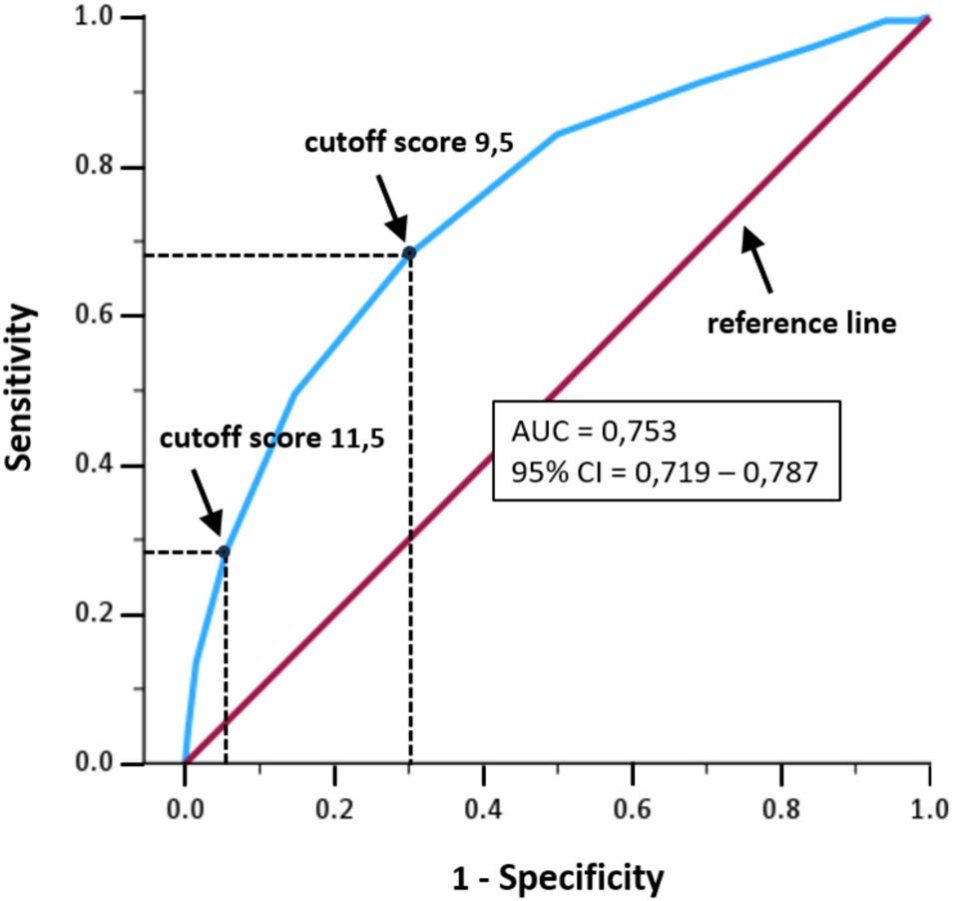
Receiver operating characteristic (ROC) curve illustrating the performance of the complex score-based model, classifying a subject as hypo- or normonatremic. Sensitivity is plotted against 1-Specificity for different cutoff model scores for hyponatremia. A total risk score above the cutoff (positive result) classifies a subject as hyponatremic; a score below the cutoff (negative result) indicates normonatremia. The area under the curve (AUC) quantifies model performance: an AUC of 0.5 (below the reference line) indicates random classification; a larger AUC indicates that the model has predictive qualities. Sensitivity (true positive rate) is the proportion of hyponatremic patients with scores above the cutoff, thus correctly classified as hyponatremic. 1-Specificity (false positive rate) is the proportion of normonatremic patients with scores above the cutoff, thus incorrectly classified as hyponatremic

Table 2 shows that female sex, older age, lower BMI, polypharmacy, and the use of PPIs, thiazide diuretics, ACE inhibitors, angiotensin-2 antagonists, thyroid preparations, and antiepileptics were associated with a higher risk of hyponatremia. Conversely, the use of vitamin K antagonists, dihydropyridines, statins, antipsychotics, and antidepressants were associated with lower risk. Nagelkerke R square was 0.17. Figure 1 illustrates the ROC curve of the complex, score-based model. The AUC was 0.75, [95% CI 0.72–0.79], indicating that the model correctly ranks 75% of the patients as either normo- or hyponatremic. This suggests that the model performs reasonably well. Table 3 provides an overview of the diagnostic values of the complex model for different cutoff scores for hyponatremia. A cutoff score of 9.5 (maximal Youden’s Index) provides the best balance between sensitivity and specificity. Of the 745 patients scoring 10 or higher, 29% (positive predictive value (PPV)) were correctly identified as having hyponatremia, and 92% (negative predictive value (NPV)) as normonatremic. When a cutoff of 11.5 was applied, 53% of the 175 patients scoring 12 or higher were accurately identified as hyponatremic. Thus, a higher cutoff of 11.5 enhances prediction accuracy, but reduces the ability to correctly identify hyponatremia, as sensitivity decreases from 0.68 to 0.29. The simple model, derived from LASSO regression, included polypharmacy as the only predictive variable. Of the 340 patients with a score of 3, (severe polypharmacy), 36% (PPV) were correctly classified as hyponatremic and 92% as normonatremic (Table 3). Nagelkerke R square was 0.051 and the AUC was 0.64 [95% CI 0.60–0.68] (electronic supplementary material).

**Table 2:** Risk profile and risk score for hyponatremia in 2181 outpatients of a geriatric clinic, according to a complex and simple model

Independent variable	B ^a^	SE ^b^	P-value	OR ^c^	95% CI ^d^		Score ^e^
Complex model							
Constant	‒4.300	0.277	< 0.01	0.014			2
Sex ^f^	0.336	0.165	0.04	1.399	1.012-	1.934	0, 1
Age categories ^g^	0.412	0.120	< 0.01	1.509	1.194-	1.909	1, 2, 3
BMI categories ^h^	0.554	0.119	< 0.01	1.740	1.378-	2.196	1, 2, 3
Polypharmacy categories ^i^	0.593	0.132	< 0.01	1.810	1.398-	2.344	1, 2, 3
Drug group (use) ^j^							
proton pump inhibitors	0.413	0.197	0.04	1.512	1.027 -	2.225	0, 1
vitamin K antagonists	‒0.527	0.218	0.02	0.590	0.385 -	0.905	0, −1
thiazide diuretics	0.593	0.190	< 0.01	1.810	1.248 -	2.625	0, 1
dihydropyridines	‒0.489	0.211	0.02	0.613	0.405 -	0.927	0, −1
ACE inhibitors ^k^	0.730	0.182	< 0.01	2.075	1.451 -	2.966	0, 1
angiotensin-2 antagonists	0.607	0.209	< 0.01	1.836	1.219 -	2.763	0, 1
statins	‒0.535	0.185	< 0.01	0.586	0.408 -	0.842	0, −1
thyroid preparations	0.546	0.251	0.03	1.727	1.056 -	2.824	0, 1
antiepileptics	1.206	0.255	< 0.01	3.342	2.028 -	5.507	0, 2
antipsychotics	‒0.904	0.393	0.02	0.405	0.188 -	0.874	0, −2
antidepressants	‒0.491	0.215	0.02	0.612	0.402 -	0.932	0, −1
Simple model							
Constant	‒3.487	0.209	< 0.01	0.031			
Polypharmacy categories	0.717	0.097	< 0.01	2.047	1.693 -	2.467	1, 2, 3

^a^ regression coefficient beta; ^b^ standard error; ^c^ odds ratio; ^d^ 95% confidence interval; ^e^ predictor score calculated by dividing the regression coefficient by 0.5,rounding the quotients up to the nearest integer, and adding 11 to the constant to make the minimal total model score 1; ^f^ male = 0, female = 1; ^g^ age (yrs) categories: < 76 = 0, 76–82 = 1. > 82 = 2; ^h^ body mass index (kg/m^2^) categories: > 27 = 1, 21–27 = 2, < 21 = 3; ^i^ polypharmacy categories: number of ATC-coded substances used: 0–4 = 1, 5–9 = 2, ≥ 10 = 3; ^j^ no use = 0, use = 1, 2, −1, or −2; ^k^ angiotensin-converting enzyme inhibitors

**Table 3:** Diagnostic values of the different cutoffs of hyponatremia risk scores of a complex and simple risk score-based model constructed for classifying 2181 outpatients of a geriatric clinic as either normo- or hyponatremic; the cutoff scores 9.5 and 11.5 are depicted by dotted lines in the model ROC curve in Fig. 1 (and Figure 2, electronic supplementary material)

Diagnostic criterion hyponatremia		All cases ≥ cutoff		Hyponatremic cases ≥ cutoff			Normonatremic cases ≥ cutoff				Classification measures					Classification					Performance measures		
≥ Cutoff ^a^	Score range	N	(%)	N	(%)		N		(%)		Sensi-tivity ^b^		1-Speci-ficity ^c^		Youden’s Index ^d^	TP ^e^	FN ^f^	FP ^g^		TN ^h^	PPV ^i^	NPV ^j^	Accuracy ^k^
Complex Model																							
0	1—16	2181	100%	230	10.5%	1951		89.5%		1.000		1.000		0.000		230	0	1951	0		10.5%		10.5%
1.5	2—16	2180	100%	230	10.6%	1950		89.4%		1.000		0.999		0.001		230	0	1949	1		10.6%	100%	10.6%
2.5	3—16	2178	100%	230	10.6%	1948		89.4%		1.000		0.998		0.002		230	0	1945	3		10.6%	100%	10.7%
3.5	4—16	2169	100%	230	10.6%	1939		89.4%		1.000		0.994		0.006		230	0	1927	12		10.7%	100%	11.2%
4.5	5—16	2148	100%	229	10.7%	1919		89.3%		0.996		0.984		0.012		228	1	1888	31		10.8%	96.9%	12.1%
5.5	6—16	2064	100%	221	10.7%	1843		89.3%		0.996		0.941		0.055		220	1	1733	110		11.3%	99.1%	16.0%
6.5	7—16	1866	100%	210	11.3%	1656		88.7%		0.961		0.843		0.118		202	8	1396	260		12.6%	96.9%	24.7%
7.5	8—16	1560	100%	194	12.4%	1366		87.6%		0.913		0.692		0.221		177	17	945	421		15.8%	96.1%	38.3%
8.5	9—16	1169	100%	157	13.4%	1012		86.6%		0.843		0.500		0.344		132	25	506	506		20.8%	95.4%	54.6%
9.5	10—16	745	100%	114	15.3%	631		84.7%		0.683		0.301		0.381		78	36	190	441		29.0%	92.4%	69.6%
10.5	11—16	401	100%	66	16.5%	335		83.5%		0.496		0.147		0.349		33	33	49	286		39.9%	89.6%	79.4%
11.5	12—16	175	100%	31	17.7%	144		82.3%		0.287		0.056		0.231		9	22	8	136		52.5%	86.0%	82.8%
12.5	13—16	59	100%	10	16.9%	49		83.1%		0.135		0.014		0.120		1	9	1	48		65.7%	84.8%	84.1%
13.5	14—16	17	100%	2	11.8%	15		88.2%		0.043		0.004		0.040		0	2	0	15		61.8%	88.7%	88.4%
14.5	15—16	3	100%	1	33.3%	2		66.7%		0.009		0.001		0.008		0	1	0	2		89.5%	66.8%	66.9%
15.5	16	1	100%	0	0.0%	1		100%		0.004		0.000		0.004		0	0	0	1			100%	100%
17		0		0		0				0.000		0.000		0.000		0	0	0	0				
** Simple Model**																							
0	1—3	2181	100.0%	230	10.5%	1951		89.5%		1.000		1.000		0.000		230	0	1951	0		10.5%		10.5%
1.5	2—3	1841	84.4%	178	14.0%	1092		86.0%		0.774		0.560		0.214		138	40	612	480		18.4%	92.3%	48.7%
2.5	3	340	15.6%	69	20.3%	271		79.7%		0.300		0.139		0.161		21	48	38	233		35.5%	82.8%	74.7%
4.0		0	0.0%	0		0				0.000		0.000		0.000		0	0	0	0				

^a^ Score ≥ cutoff = positive result = classified as hyponatremia; score < cutoff = negative result = classified as normonatremia; ^b^ Sensitivity = true positive rate = proportion of patients with hyponatremia, correctly classified as hyponatremic (TP/(TP + FN); ^c^ 1-Specificity = false positive rate = proportion of patients with normonatremia, but incorrectly classified as hyponatremic (FP/(TN + FP); ^d^ Youden’s Index = measure of model performance (sensitivity + specificity – 1); a maximal value indicates the model performs optimal in discriminating TP from FP results; ^e^ TP = true positives = number of patients with hyponatremia with scores ABOVE the cutoff, thus correctly classified as hyponatremic; ^f^ FN = false negatives = number of patients with hyponatremia but with scores BELOW the cutoff, thus incorrectly classified as normonatremic; ^g^ FP = false positives = number of patients with normonatremia but with scores ABOVE the cutoff, thus incorrectly classified as hyponatremic; ^h^ TN = true negatives = number of patients with normonatremia with scores BELOW the cutoff, thus correctly classified as normonatremic; ^ij^ PPV = Positive predictive value = TP/(TP + FP), proportion of patients with scores ABOVE the cutoff, correctly classified as hyponatremic; ^j^ NPV = Negative predictive value = TN/(TN + FN), proportion of patients with scores BELOW the cutoff, correctly classified as normonatremic; ^k^ Accuracy = (TP + TN)/(TP + FP + TN + FN)

## Discussion

Chronic hyponatremia, a condition characterised by atypical complaints, is underrecognized in older people. To address this, we developed a simple and complex score-based model to predict chronic hyponatremia in outpatients of a geriatric clinic. The complex model included sex, age, BMI, polypharmacy, and 11 drug groups as predictors. The model demonstrated reasonably good predictive ability, correctly classifying 75% of the patients as normo- or hyponatremic.

### Predictors of hyponatremia

Female sex was associated with increased hyponatremia risk, which is consistent with previous findings [19, 20]. The positive association between age and hyponatremia, also observed in other studies [19–21], may be confounded by frailty [22]. The higher probability of hyponatremia in patients with lower BMI may be explained by frailty as well, as low body weight is a known determinant [23]. The association with polypharmacy, as described in the literature [2], could be due to the increased probability of being prescribed hyponatremia-inducing drugs. Additionally, polypharmacy may serve as a proxy for the number of comorbidities, another determinant of frailty [23].

Several associations with individual drug groups have been reported [4, 15]. Hyponatremia is common among users of ***thiazide diuretics***, occurring in approximately 11% of geriatric patients and 14% in primary care [4, 15]. Underlying mechanisms include sodium loss due to inhibited sodium reabsorption and water excretion in the distal nephron, and increased fluid intake (thirst) [24]. A lower prevalence of hyponatremia in ***statin*** users was observed in case–control studies in the general Swedish population and in a Thai cohort [19, 25]. One possible explanation is a reduced fractional excretion of sodium in the short-term and long-term preservation of kidney function [26, 27]. Another is the higher BMI of statin users [28], a known determinant of frailty [23]. The inverse association of ***dihydropyridine calcium-antagonists*** contrasts with a positive association reported in an observational and a case study [29, 30]. The inverse association with ***vitamin K antagonists*** remains unexplained. Consistent with our findings, long-term use of *a****ngiotensin-II receptor antagonists*** and ***ACE inhibitors*** has been associated with hyponatremia in older people in Italy and Sweden [2, 31]. This may reflect confounding by indication, as heart failure as well as hypertension increase antidiuretic hormone (ADH) production and stimulate the renin–angiotensin–aldosterone system (RAAS), both of which promote water reabsorption, thus increasing hyponatremia risk. Impaired kidney function may further compromise sodium homeostasis [32]. Certain ***antiepileptics***, particularly carbamazepine, are known to cause the syndrome of inappropriate antidiuretic hormone secretion (SIADH), leading to water retention and hypotonic hyponatremia [33]. This supports the higher probability of hyponatremia in our antiepileptic users. Although we expected a similar association with ***antidepressants*** and ***antipsychotics*,** which can also induce SIADH [34, 35], we found the opposite. This may reflect the differential impact of treatment duration on sodium levels. Risk of hyponatremia is highest during the first month of **antidepressant** therapy, with 75% of cases occurring in this period [36, 37]. After 3–6 months, risk declines to that of non-users [38–40]. Long-term use may further lower risk due to weight gain [41], which is associated with reduced hyponatremia risk. As most patients had chronic conditions, they were likely long-term users. A similar explanation may apply to the inverse association with ***antipsychotics*** [42, 43]. The association between ***thyroid agents*** and hyponatremia may reflect confounding by disease, as hypothyroidism can cause hyponatremia. Suggested mechanisms include increased body weight, reduced cardiac output due to bradycardia (lowering GFR and raising ADH), reduced atrial natriuric peptide (increasing water retention), and decreased sodium reabsorption [44]. Finally, a positive association between ***proton pump inhibitors*** and hyponatremia was found in a small prospective study [45], but its statistical methods used have been debated [46, 47]. A recent case–control study found an increased occurrence of hyponatremia-related hospitalization within 90 days of starting a PPI. Longer PPI use was not associated with this risk, and risk of hyponatremia without hospitalization was not studied [48]. As PPIs are linked to polypharmacy, the positive association with hyponatremia may reflect the hyponatremia-inducing effects of one or more other drugs used together with PPIs, or comorbidities related with frailty. Another explanation is the potential of PPIs to induce hypomagnesemia, a known risk factor of hyponatremia [49].

### Other drug-including models predicting hyponatremia

To our knowledge, two studies have reported on models predicting hyponatremia that included drug use [19, 50]. The first, developed for hypertensive patients based on a case control study, demonstrated good discriminative performance of the model (AUC 0.8153, se 0.0138). Predictors identified in this model, which were also found in our study, included sex, age, and the use of statins and benzodiazepines. However, the model was developed for patients already using thiazides and was not externally validated [19]. The second model, based on a prospective study predicted hyponatremia after brain surgery (AUC 0.742, se = 0.04). However, this model was designed to detect short-term occurrence of hyponatremia, the model was not externally validated, and included medications that were hardly prescribed in our study population [50].

### Strengths, limitations

To our knowledge, this is the first study to develop regression models to describe and predict hyponatremia in outpatients of a geriatric clinic. Given that our cases were likely patients with chronic hyponatremia, the models could be valuable to detect chronic hyponatremia and enhance understanding of this condition in outpatients of a geriatric clinic with atypical complaints. We used non-invasive, easy to measure predictors, focusing on determinants of drug use. Accurate recording of drug and supplement use during consultation, conversion of these data into ATC codes, standardized measurement of height and length, and measurement of sodium concentrations by the hospital lab strengthen the study. In addition to the complex model developed with stepwise backward logistic regression, we constructed a simpler model using LASSO regression. Although LASSO regression was applied to reduce overfitting, the number of hyponatremic events per predictor in the complex logistic model exceeded the recommended minimum of 10, as suggested by Peduzzi et al. (1996) [51]. We validated the confidence intervals of the regression coefficients in both models using bootstrapping (internal validation). The use of LASSO regression, which reduces the number of predictors, not only helps preventing overfitting but also improves the model’s interpretability [52].

However, reducing predictors through Lasso regression may introduce bias and lead to underfitting [52]. Furthermore, the predictive performance of the simple model was limited compared to the complex model. Another concern is selection bias, as sodium concentrations were measured in only 81% of new visitors of the outpatient clinic. Additionally, we excluded patients using antidiabetic medications to minimize the number of cases with secondary hyponatremia, as hyperglycemia affects sodium values. Although we could have included diabetic patients and adjusted sodium concentrations for hyperglycemia, only one study in emergency patients supports this approach [6]. Among the hyponatremic cases, pseudohyponatremia due to elevated lipid or protein levels, or pseudo-normonatremia due to low protein levels, may have occurred, as sodium was measured by indirect potentiometry. However, only extreme lipid or protein levels would obscure actual sodium values [53]. As we had no data on duration of drug use, we could not substantiate our interpretation of the inverse associations with antidepressants and antipsychotics. Additionally, the cross-sectional design only allows for speculation about causal relationships with predictors. Smoking status, potentially contributing to hyponatremia [49], could not be assessed due to the inaccurate registration in patient files. Although sex-related differences in pharmacokinetics or pharmacodynamics of certain drugs have been described [54], we did not stratify our analyses by sex due to the limited number of hyponatremic cases. Finally, the models were internally validated via bootstrapping; external validation was not performed.

## Conclusion

We developed a drug-based model based on drug use and other easily assessable, non-invasive variables to predict the risk of hyponatremia in outpatients of a geriatric clinic. Timely recognition may help prevent inappropriate treatments and associated harms in undiagnosed cases. Given its potential clinical relevance, we recommend further refinement of the model, including the development of separate models for men and women, and the investigation of smoking status as an extra predictor. Additionally, these models should be externally validated in diverse populations of older individuals.

## Supplementary Information

Below is the link to the electronic supplementary material.

Supplementary file1 (DOCX 119 KB)

## Data Availability

The patient data are owned by Gelderse Vallei Hospital in Ede, The Netherlands, and are not publicly accessible. If access is required, please contact the corresponding author.
