# Supplementary material for: A drug-based model to predict hyponatremia in outpatients of a geriatric clinic
Source: Eur J Clin Pharmacol. 2025 Aug 1;81(10):1507–15. doi: 10.1007/s00228-025-03890-y (PMC12443923; doi:10.1007/s00228-025-03890-y)
Supplement: Supplementary file 1 — Supplementary file1 (DOCX 119 KB) [file 228_2025_3890_MOESM1_ESM.docx]

**Title manuscript**

A drug-based model to predict chronic hyponatremia in geriatric outpatients

**Authors**

Anne Claire B. van Orten-Luiten, Elske M. Brouwer-Brolsma, André Janse, Renger F. Witkamp

**Supplementary Information**

| **Table 4** Frequency of use of 27 specific drug groups and dietary supplements in 2181 geriatric outpatients | | | | | | | | | | | | | | |
| --- | --- | --- | --- | --- | --- | --- | --- | --- | --- | --- | --- | --- | --- | --- |
| **ATC-coded substance** | | | | | **ATC code ^b^** | **Use in**  **total population**  **(N=2181)** | | |  | **Use if**  **hyponatremia**  **(N=230)** | |  | **Use if**  **normonatremia**  **(N=1951)** | |
|  |  |  |  |  |  | **n** | **%** | **rank** |  | **n** | **%** |  | **n** | **%** |
|  | | | | |  |  |  |  |  |  |  |  |  |  |
| Proton pump inhibitors | | | | | A02BC | 1092 | 50.1 % | 1 |  | 165 | 71.7 % |  | 927 | 47.5 % |
| Osmotically acting laxatives | | | | | A06AD | 330 | 15.1 % | 12 |  | 51 | 22.2 % |  | 279 | 14.3 % |
| Multivitamins and minerals | | | | | A11A | 184 | 8.4 % | 19 |  | 23 | 10.0 % |  | 161 | 8.3 % |
| Vitamin D | | | | | A11CC | 629 | 28.8 % | 3 |  | 84 | 36.5 % |  | 545 | 27.9 % |
| Calcium | | | | | A12AA | 331 | 15.2 % | 11 |  | 51 | 22.2 % |  | 280 | 14.4 % |
| Potassium | | | | | A12BA | 164 | 7.5 % | 20 |  | 29 | 12.6 % |  | 135 | 6.9 % |
| Vitamin K antagonists | | | | | B01AA | 320 | 14.7 % | 13 |  | 32 | 13.9 % |  | 288 | 14.8 % |
| Platelet aggregation inhibitors | | | | | B01AC | 700 | 32.1 % | 2 |  | 96 | 41.7 % |  | 604 | 31.0 % |
| Cardiac therapy | | | | | C01 | 195 | 8.9 % | 18 |  | 26 | 11.3 % |  | 169 | 8.7 % |
| Thiazide diuretics | | | | | C03AA | 368 | 16.9 % | 9 |  | 73 | 31.7 % |  | 295 | 15.1 % |
| Loop diuretics | | | | | C03CA | 230 | 10.5 % | 16 |  | 27 | 11.7 % |  | 203 | 10.4 % |
| Potassium-sparing diuretics | | | | | C03D | 132 | 6.1 % | 25 |  | 26 | 11.3 % |  | 106 | 5.4 % |
| Selective beta blockers | | | | | C07AB | 585 | 26.8 % | 4 |  | 77 | 33.5 % |  | 508 | 26.0 % |
| Dihydropyridines | | | | | C08CA | 348 | 16.0 % | 10 |  | 36 | 15.7 % |  | 312 | 16.0 % |
| ACE inhibitors ^c^ | | | | | C09AA | 460 | 21.1 % | 6 |  | 71 | 30.9 % |  | 389 | 19.9 % |
| Angiotensin-2 antagonists | | | | | C09CA | 290 | 13.3 % | 15 |  | 50 | 21.7 % |  | 240 | 12.3 % |
| Statins | | | | | C10AA | 560 | 25.7 % | 5 |  | 52 | 22.6 % |  | 508 | 26.0 % |
| Thyroid preparations | | | | | H03A | 138 | 6.3 % | 23 |  | 27 | 11.7 % |  | 111 | 5.7 % |
| NSAIDs ^d^ | | | | | M01A | 156 | 7.2 % | 21 |  | 17 | 7.4 % |  | 139 | 7.1 % |
| Bisphosphonates | | | | | M05BA | 155 | 7.1 % | 22 |  | 22 | 9.6 % |  | 133 | 6.8 % |
| Opioid analgetics | | | | | N02A | 123 | 5.6% | 26 |  | 20 | 8.7% |  | 103 | 5.3% |
| Anilides (paracetamol) | | | | | N02BE | 314 | 14.4 % | 14 |  | 54 | 23.5 % |  | 260 | 13.3% |
| Antiepileptics | | | | | N03 | 119 | 5.5 % | 27 |  | 29 | 12.6 % |  | 90 | 4.6 % |
| Antipsychotics | | | | | N05A | 136 | 6.2 % | 24 |  | 8 | 3.5 % |  | 128 | 6.6 % |
| Benzodiazepines ^e^ | | | | | N05 ^e^ | 399 | 18.3 % | 7 |  | 58 | 25.2 % |  | 341 | 17.5 % |
| Antidepressants | | | | | N06A | 383 | 17.6 % | 8 |  | 35 | 15.2 % |  | 348 | 17.8 % |
| Adrenergic inhalants | | | | | R03A | 202 | 9.3 % | 17 |  | 26 | 11.3 % |  | 176 | 9.0 % |
| Any ATC-coded supplement ^f^ | | | | | multiple | 1105 | 50.7 % |  |  | 143 | 62.2% |  | 962 | 49.3% |
|  |  |  |  |  | | | | | | | | | | |
| ^a^ estimated glomerular filtration rate (ml/min/1.73m^2^); ^b^ Anatomical Therapeutic Chemical classification code; ^c^ angiotensin-converting enzyme inhibitors; ^d^ non-steroid antiinflammatory drugs; ^e^ N05BA, NO5CD, NO5CF; ^f^ use of any ATC-coded dietary supplement: A02AA, A11, A12, B03A-, B03B-, A16AA01, C10AX06, M01AX05, N05C, N06DX02; this variable was not included as a candidate predictor | | | | | | | | | | | | | | |


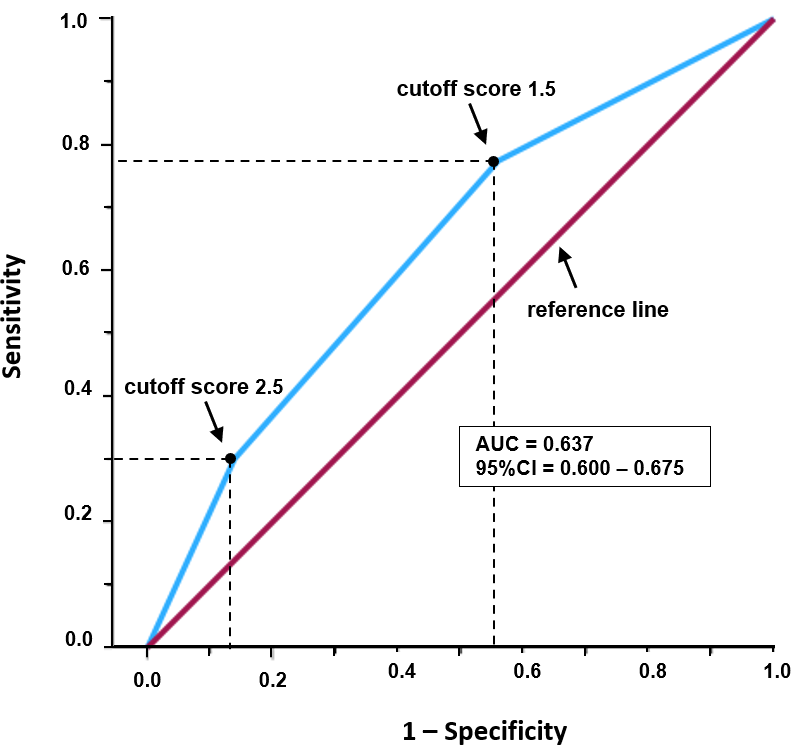


| \| **Figure 2**  Receiver operating characteristic (ROC) curve illustrating the performance of the simple score-based model, classifying a subject as hypo- or normonatremic. Sensitivity is plotted against 1-Specificity for different cutoff model scores for hyponatremia. A total risk score above the cutoff (positive result) classifies a subject as hyponatremic; a score below the cutoff (negative result) indicates normonatremia. The area under the curve (AUC) quantifies model performance: an AUC of 0.5 (below the reference line) indicates random classification; a larger AUC indicates that the model has predictive qualities. Sensitivity (true positive rate) is the proportion of hyponatremic patients with scores above the cutoff, thus correctly classified as hyponatremic. 1-Specificity (false positive rate) is the proportion of normonatremic patients with scores above the cutoff, thus incorrectly classified as hyponatremic. \| \| --- \| |
| --- | --- |
